# Progressive Weakness in Adulthood: Lipid Storage Myopathy With Suspected Sertraline-Associated Etiology

**DOI:** 10.7759/cureus.99645

**Published:** 2025-12-19

**Authors:** Jaclyn Feely, Natalie Saliba, NdukeAbasi Nze, Jeffrey Nguyen

**Affiliations:** 1 Medicine, Alabama College of Osteopathic Medicine, Dothan, USA; 2 Medicine, Edward Via College of Osteopathic Medicine, Auburn, USA; 3 Medicine, University of Alabama at Birmingham Heersink School of Medicine, Birmingham, USA; 4 Neurology, Neurology Institute of Huntsville, Huntsville, USA

**Keywords:** acute muscle weakness, fatty acid oxidation, lipid metabolism, myopathy, sertraline

## Abstract

Lipid storage myopathies (LSMs) are uncommon metabolic disorders that are not always considered during routine evaluations for weakness. We describe a 51-year-old woman with progressive proximal weakness. The patient's initial studies included electromyography (EMG), spine imaging, and serologies, all of which were unrevealing. A muscle biopsy revealed lipid accumulation, and genetic testing suggested a deficiency in multiple acyl-CoA dehydrogenase (MADD) or a defect in the riboflavin transporter. Riboflavin supplementation was recommended, and a sertraline taper was initiated after concern that prolonged selective serotonin reuptake inhibitor (SSRI) exposure might be contributing to her mitochondrial dysfunction. This case demonstrates the importance of considering metabolic myopathies in patients with unexplained progressive weakness, particularly when conventional diagnostics are inconclusive.

## Introduction

Lipid storage myopathies (LSMs) are a group of metabolic muscle disorders that cause the abnormal accumulation of lipids in skeletal muscle fibers due to defects in fatty acid oxidation. Fatty acid oxidation disorders present with a broad spectrum of clinical manifestations, ranging from exercise intolerance and progressive muscle weakness to multiorgan involvement [1,2].

Acquired LSMs may arise from medications (e.g., statins, corticosteroids), nutritional deficiencies (carnitine, riboflavin), endocrine disorders (e.g., hypothyroidism), or mitochondrial toxicity from chronic illness or drugs [3]. Genetic subtypes include multiple acyl-CoA dehydrogenase deficiency (MADD), primary carnitine deficiency, neutral lipid storage disease, and carnitine palmitoyltransferase II deficiency [4-6]. MADD is particularly notable because late-onset forms may be responsive to riboflavin supplementation [4-6]. Recognition of such defects is critical, as early riboflavin supplementation significantly improves outcomes [6].

This report presents a middle-aged woman with progressive proximal weakness whose biopsy and genetic studies suggested MADD or a riboflavin transport disorder. She had prolonged exposure to sertraline, which is not typically associated with metabolic myopathies. There are other case reports to suggest that selective serotonin reuptake inhibitors (SSRIs) may, in rare cases, contribute to mitochondrial dysfunction [7]. This case emphasizes the need for broad differential diagnosis of unexplained weakness and vigilance regarding drug-induced myopathies.

## Case presentation

A 51-year-old woman with hypertension, diabetes mellitus, anxiety, and depression presented with progressive weakness that worsened with exertion and improved with rest. Associated symptoms included numbness, tingling, and intermittent involvement of the upper extremities. She denied cranial nerve symptoms, dysphagia, or dyspnea at onset.

Her medications included: losartan 100 mg daily, labetalol 100 mg twice daily, nifedipine ER 60 mg daily, pravastatin 40 mg daily, bupropion 100 mg twice daily, meloxicam 7.5 mg daily, eszopiclone 3 mg nightly, sertraline 100 mg daily, hydralazine 50 mg twice daily, clonazepam 0.5 mg daily, glipizide ER 10 mg daily, and gabapentin 600 mg three times daily.

Surgical history included hysterectomy, knee replacement, schwannoma removal, and cerebrospinal fluid (CSF) shunt placement. Family history was notable for amyotrophic lateral sclerosis (ALS) in her paternal grandfather. She did not smoke, drink, or use illicit drugs.

Initial neurologic examination was unremarkable aside from subjective weakness. Routine labs showed aldolase 7.5 U/L and creatine kinase 359 U/L. Electromyography (EMG)/nerve conduction study (NCS) revealed only mild right carpal tunnel syndrome without evidence of myopathy. MRI and CT of the spine showed degenerative changes without significant pathology. She was referred to physical therapy but reported worsening weakness, eventually requiring a wheelchair. A hospitalization for a urinary tract infection exacerbated her weakness, and over a few months, the patient developed dysarthria, dysphagia, and daily migraines, for which topiramate was started.

At a tertiary referral center, muscle biopsy demonstrated abnormal lipid accumulation. Genetic testing suggested MADD or a riboflavin transporter disorder. Primary carnitine deficiency was excluded, given normal free carnitine levels. Riboflavin supplementation was recommended, and sertraline tapering was initiated after concern that prolonged SSRI exposure might be contributing to her mitochondrial dysfunction. Whole genome sequencing was initiated for further clarification.

## Discussion

This case highlights the diagnostic challenges of LSMs, particularly when symptoms are nonspecific and early investigations are unrevealing [8,9]. LSMs are known to present with exercise intolerance and progressive weakness with normal initial studies [10]. Due to the lack of improvement, an advanced evaluation at a tertiary center was pursued, and a muscle biopsy ultimately revealed the metabolic etiology. Biopsy of the muscle remains the confirmatory test for lipid myopathy and is critical when noninvasive studies are inconclusive [11].

**Figure 1:**
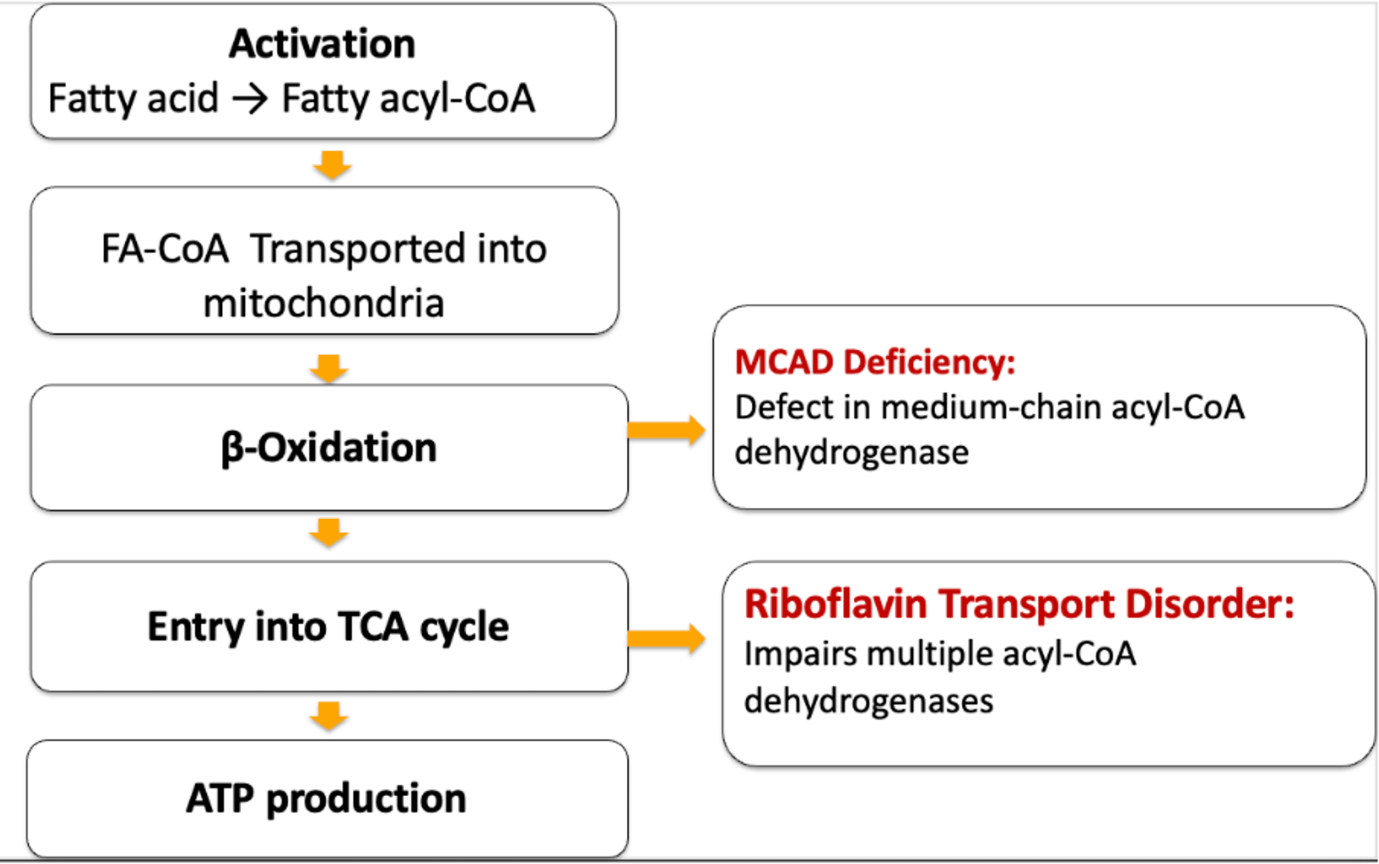
Simplified schematic of the fatty acid oxidation pathway. Sites of impairment in MADD and riboflavin transporter defects are highlighted. Created by the authors based on information from [16]. MADD: Multiple acyl-CoA dehydrogenase deficiency

The differential diagnosis in this case initially included inflammatory myopathies, neuromuscular junction disorders, and neurodegenerative diseases. The absence of myopathic EMG findings with the normal muscle enzymes and negative autoimmune serologies made these less likely. Ultimately, the combination of a muscle biopsy showing lipid accumulation and genetic testing suggesting MADD or a riboflavin transporter defect provided diagnostic clarity. Figure 1 demonstrates the steps in the metabolic pathway where MADD and riboflavin transporter defects disrupt normal function. Early suspicion of a metabolic myopathy, followed by targeted genetic analysis, has been shown to shorten diagnostic delay and facilitate timely initiation of vitamin or cofactor therapy [15,16]. Overall, this case highlights that LSMs can mimic more common neuromuscular disorders and that a high index of suspicion is required when standard studies are unrevealing. Early recognition of potentially reversible factors, including vitamin-responsive forms and medication-associated mitochondrial toxicity, can significantly improve patient outcomes [17]. 

A potential association between sertraline and mitochondrial dysfunction has been suspected in the literature. SSRIs are widely prescribed and generally well tolerated, but isolated reports have linked them to myopathy and impaired oxidative metabolism. Although the mechanism remains unclear, in vitro studies have demonstrated that SSRIs can impair mitochondrial respiratory chain complexes and reduce ATP production, leading to increased oxidative stress [12]. A few case reports have described SSRI-associated myopathies or mitochondrial toxicity [13]. Given the temporal association and absence of alternative acquired causes, sertraline may have contributed to an acquired mitochondrial dysfunction that exacerbated an underlying genetic predisposition demonstrated in [14]. Several medications are noted to cause similar effects and are described in Table 1.

**Table 1:** Several medications have been implicated in acquired LSMs through various mechanisms Common medications linked to myopathies and their mechanisms. Created by the authors based on information from [17]. LSM: Lipid storage myopathy; NRTI: Nucleoside reverse transcriptase inhibitor

Class	Examples	Mechanism Leading to Myopathy
Statins	Atorvastatin	Mitochondrial dysfunction and apoptosis
Glucocorticoids	Prednisone, Dexamethasone	Protein catabolism and muscle fiber atrophy (type II fibers)
Antimalarials	Chloroquine, Hydroxychloroquine	Lysosomal dysfunction → impaired autophagy and accumulation of toxic metabolites
Colchicine	Colchicine	Impaired transport and autophagy → vacuolar myopathy
Antiretrovirals (NRTIs)	Zidovudine, Stavudine	Impaired oxidative phosphorylation
Amiodarone	Amiodarone	Abnormal phospholipid accumulation
Alcohol	Ethanol	Direct toxicity, oxidative stress, and nutritional deficiency
Cytotoxic Agents	Vincristine, Cyclophosphamide	Microtubule disruption or direct toxicity
Fibrates	Gemfibrozil, Fenofibrate	Interference with fatty acid oxidation → mitochondrial dysfunction

## Conclusions

This case illustrates an atypical presentation of LSM with possible sertraline-related mitochondrial dysfunction. Clinicians should maintain suspicion for metabolic myopathies when standard evaluations are unrevealing and pursue biopsy and genetic testing when appropriate. Awareness of rare, medication-induced myopathies is essential, particularly in patients with long-standing use of drugs not classically linked to muscle toxicity. Early recognition and multidisciplinary management can improve outcomes in rare disorders such as LSM.
